# Model of Biological Quantum Logic in DNA

**DOI:** 10.3390/life3030474

**Published:** 2013-08-02

**Authors:** F. Matthew Mihelic

**Affiliations:** Department of Family Medicine, University of Tennessee Graduate School of Medicine, 1924 Alcoa Highway, U-67, Knoxville, TN 37920, USA; E-Mail: fmihelic@utmck.edu; Tel.: +1-865-305-9352; Fax: +1-865-305-6532

**Keywords:** quantum logic, DNA, quantum coherence

## Abstract

The DNA molecule has properties that allow it to act as a quantum logic processor. It has been demonstrated that there is coherent conduction of electrons longitudinally along the DNA molecule through pi stacking interactions of the aromatic nucleotide bases, and it has also been demonstrated that electrons moving longitudinally along the DNA molecule are subject to a very efficient electron spin filtering effect as the helicity of the DNA molecule interacts with the spin of the electron. This means that, in DNA, electrons are coherently conducted along a very efficient spin filter. Coherent electron spin is held in a logically and thermodynamically reversible chiral symmetry between the C2-endo and C3-endo enantiomers of the deoxyribose moiety in each nucleotide, which enables each nucleotide to function as a quantum gate. The symmetry break that provides for quantum decision in the system is determined by the spin direction of an electron that has an orbital angular momentum that is sufficient to overcome the energy barrier of the double well potential separating the C2-endo and C3-endo enantiomers, and that enantiomeric energy barrier is appropriate to the Landauer limit of the energy necessary to randomize one bit of information.

## 1. Introduction

In 1944 Schrödinger intuited that the quantum mechanical processes inherent in a biological system must take place in the controlling genetic material of the system, and that that genetic material must therefore be in the form of some sort of “aperiodic crystal” [1]. In 1953, that genetic “aperiodic crystal” was characterized by Watson and Crick as the double helix of the DNA molecule. Watson and Crick were heavily influenced by Schrödinger, but the quantum mechanical rationale that formed the basis of that influence was forgotten. The last sixty years have brought a huge increase in information regarding quantum mechanics and nucleic acids, and so a viable model of the mechanism of quantum logic in DNA can now be described that is consistent with Schrödinger’s intuited rationale.

In brief, the DNA molecule has properties that allow it to act as a quantum logic processor. An electron or its quantum state can be coherently conducted or quantum teleported longitudinally along the coherence provided by the pi stacking of the nucleotide bases of the DNA molecule [2]. As an electron or its quantum state is conducted or teleported longitudinally along the DNA molecule it is simultaneously subject to an electron spin filtration effect that is brought about by interaction of the helicity of the DNA molecule with the spin of the electron [3], and this provides the means for selective deposition of an individual electron, or the reading of an individual electron state, into a specific individual nucleotide quantum gate as determined by the electron spin direction and the coherence distance along the DNA molecule. Quantum logical operations in DNA occur via a quantum logic gate capability in each nucleotide that is provided by a logically and thermodynamically reversible Szilard engine function [4] of the deoxyribose moiety through which coherent electron spin is held in an enantiomeric symmetry between the C2-endo and C3-endo enantiomers in the nucleotide. The symmetry break that provides for quantum decision in the system is determined by the spin direction of an electron that has an orbital angular momentum that is sufficient to overcome the energy barrier of the double well potential separating the C2-endo and C3-endo deoxyribose enantiomers. The energy barrier of that double well potential [5] is appropriate to the Landauer limit of the energy necessary to randomize one bit of information, thereby enabling chirality determination by electron spin at an energy level appropriate to quantum logical operation. The individual nucleotide quantum gates are held in coherent concatenation through the pi stacking interactions of the nucleotide bases in the DNA molecule, and longitudinal coherence distances can be affected by deoxyribose enantiomeric selection that can bring about a change in the orientation of the nucleotide’s base by a change in the N-glucosidic bond angle. The crystalline nature of the DNA molecule allows for extended temporal coherence of the system through its precisely designed nanospace which limits the degrees of freedom upon which entropic factors such as temperature or solvation can have any effect, and within which inherently fault-tolerant topological quantum logic operations can take place.

Certain conditions should exist for quantum logic processes to occur. A quantum logic system must have a pathway of coherence by which qubits of superimposed information can be conducted and held in superposition, as well as a mechanism of quantum decision by which such coherence can be broken, and also a quantum gate by which those qubits of superimposed information can interact coherently. These components of a quantum logic system are properties of DNA and as such provide for the quantum logic function of the DNA molecule.

Coherent conduction of electrons along the pi stacking of the nucleotide base pairs in DNA has been demonstrated by several investigators [2,6], and this would allow for the information contained in the nucleotide base pair sequence to be held coherently among those nucleotides that are involved in that coherent conduction. In addition it has been demonstrated that the helicity of the DNA molecule provides a very efficient electron spin filtering effect upon electrons that move longitudinally along the DNA molecule [3]. This means that in DNA, electrons are coherently conducted along an electron spin filter that can discriminate between their spin states, and thereby selectively filter electrons of a particular spin direction out of the conduction pathway if they have travelled far enough along the molecule to generate enough of an electromotive force as would be necessary for such selection [7,8]. This would allow for the filtration of an electron or its state at a selective point that is determined by its spin state and by the distance that it is coherently conducted along the DNA molecule, and that selective point is an individual nucleotide that functions as a quantum gate.

A Szilard engine uses information to convert a particle’s momentum into useful work, and it has been theoretically demonstrated how a Szilard engine can be physically constructed out of the information by which it functions in such a manner as to make it simultaneously both logically and thermodynamically reversible, and therefore function as a quantum logic gate [4]. DNA is a polymer that physically constitutes the information that defines the concept and function of the biological system in which it is found, and since the DNA molecule is the physicality of the genetic information by which a biological system functions, it should be considered as potentially possessing such logically and thermodynamically reversible quantum logic gate function if Szilard engine function were to take place within it [9,10]. Such reversible Szilard engine function can be theoretically shown to take place in each nucleotide of a DNA molecule, allowing nucleotides to function as concatenated quantum gates by which qubits of electron spin can coherently interact. Thus it is that a theoretical reversible Szilard engine quantum logic gate function in DNA combines with the demonstrated DNA properties of coherent electron conduction and electron spin filtration to effect quantum logic function within and throughout the DNA molecule.

## 2. Furanose Ring “Pseudorotation”

The quantum gate function that enables quantum logic function in DNA occurs in the deoxyribose moiety of the DNA nucleotide. Deoxyribose is derived from the furanose molecule and is a cyclic 5-carbon sugar that has a “puckered” ring structure. The ring structure exhibits a “pseudorotation” around a series of conformational changes in that “sugar pucker” and the energy barriers between the conformational changes are relatively low. Of special significance are the conformations of the C2-endo and C3-endo enantiomers that are separated by an energy barrier of 0.6 kcal/mole [5]. (Note that this paper will deal mainly with the C2-endo and C3-endo enantiomers, but that other conformations of furanose ring pseudorotation not discussed here may also have relevance to the quantum logic processes of DNA). The energy barrier between these two enantiomers is low enough to allow easy conformational shifting back and forth between them, and when such a shift occurs in the deoxyribose ring of the DNA molecule it effects other significant conformational changes in the nucleotide that it is a part of. Specifically, when the C2-endo enantiomer shifts to the C3-endo enantiomer there is a rotation around the C3-C4 bond that can bring about a shift in the orientations of the C3-carbon and the C5-carbon, and this brings about a local conformational shift in the DNA phosphate backbone that changes the orientation and distance between respective attached phosphorus atoms. At the same time this enantiomeric shift between C2-endo and C3-endo can bring about a significant change in the angle of the N-glucosidic bond that changes the orientation of the nucleotide base that is attached to the C1-carbon of the deoxyribose portion of the nucleotide. So in the deoxyribose moiety of the DNA nucleotide a low-energy conformational change between the C2-endo and C3-endo enantiomers can bring about significant changes in the conformation of a portion of the phosphate backbone and also in the orientation of the nucleotide base [11].

## 3. A Quantum Gate in the Deoxyribose Moiety

There is effectively a symmetry or a type of quantum equilibrium that exists across what amounts to a double well potential between the C2-endo and C3-endo deoxyribose conformations, and the enantiomeric selection between these two conformational isomers is ultimately determined by electron spin. Electron spin is known to determine the chirality of stereoisomers due to spin-orbit coupling at a chiral center, and so it would follow that somewhere in the nucleotide there is a covalent bond at a chiral center that would determine this enantiomeric conformation of the deoxyribose ring. The likely position for such a deoxyribose enantiomeric chirality-determining covalent bond in the nucleotide is the covalent bond between the C2-carbon and the C3-carbon, which is situated between the obvious chiral center at C3 and the not-so-obvious atropisomeric chiral center at C2. The orbital angular momentum of an electron can provide the energy necessary to overcome the relatively low energy barrier between the C2-endo and C3-endo conformations, and the direction of the electron spin can provide the direction to determine the chiral selection between the two enantiomers. This conversion of a particle’s orbital angular momentum and spin direction into the useful work of physical selection between two enantiomers effects a symmetry break, and is essentially the function of a Szilard engine [12] that is taking place within the physicality of the information that is DNA, because the information by which such a nucleic acid Szilard engine (NASE) functions can be held coherently within and throughout the base pair sequence of the DNA molecule.

While the traditionally theorized Szilard engine is not thermodynamically reversible, it has been theoretically demonstrated that a Szilard engine will be logically and thermodynamically reversible if it’s mechanism is bounded in part by the physicality of the information by which it functions in such a manner that that information is available simultaneously to both sides of the bounding wall of the engine, and that such a reversible Szilard engine would therefore be capable of function as a quantum gate [4]. The enantiomeric shift between the C2-endo and C3-endo conformations in the deoxyribose moiety of the DNA molecule occurs because electron orbital angular momentum (along with electron spin direction) is converted into the useful work of enantiomeric selection, and this is essentially Szilard engine function because the essence of Szilard engine function is that information is used to convert particle momentum into the useful work of a symmetry break [12]. Nucleotides perform this symmetry-breaking function of chirality determination by utilizing the base pair sequence information that is coherently held within and between them due to pi stacking interactions of aromatic nucleotide bases, and because that information physically forms part of its boundary, that Szilard engine mechanism is logically and thermodynamically reversible and therefore functions as a quantum gate.

**Figure 1:**
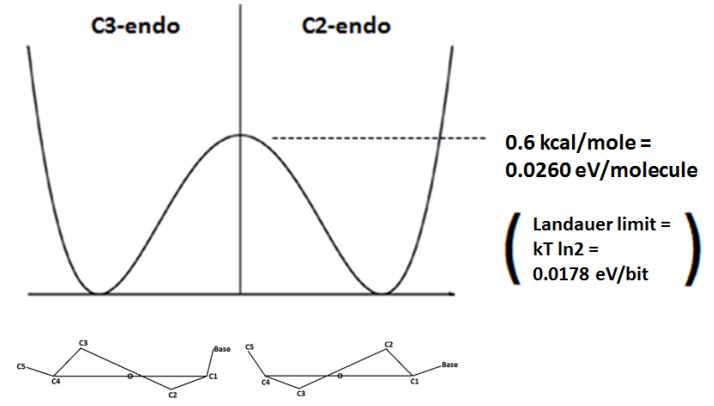
When rotation around the C3–C4 bond is constrained a double well potential exists in which the C3-endo and C2-endo enantiomers are separated an energy barrier of 0.6 kcal/mole, and this energy barrier is appropriately slightly more than the Landauer limit of the energy necessary to randomize one bit of information. This energy barrier can be overcome by the energy associated with the orbital angular momentum of a coherently conducted electron that has longitudinally passed through the helical spin filter of the DNA molecule. With the energy barrier overcome and thereby essentially “randomized”, the spin direction of that electron can determine the enantiomeric selection/decision between C3-endo and C2-endo.

If the C3-C4 bond is constrained in the deoxyribose ring of DNA, the C2-endo and C3-endo enantiomeric conformations are separated by an energy barrier of 0.6 kcal/mole in what can be considered to be a double well potential [5]. It is of note that this 0.6 kcal/mole mathematically converts to approximately 0.0260 eV per molecule, and this is significant because it is similar to, but appropriately more than 0.0178 eV per bit, which is the so-called “Landauer limit” (kT ln2) of the energy necessary to erase one bit of information at 25 °C. Therefore, the energy barrier represented by the double well potential between the C2-endo and C3-endo enantiomeric conformations can be overcome by an electron’s orbital angular momentum that is appropriate to the Landauer limit of the energy necessary to randomize one bit of information, and once that bit is randomized the electron spin direction can determine the enantiomeric conformation. So the energy necessary to break the symmetry of what is essentially a double well potential set up between the C2-endo and C3-endo enantiomeric conformations, is appropriate to the theoretical energy necessary to randomize one bit of information, which enables very efficient conservation and manipulation of information as would be appropriate to the function of a quantum gate (Figure 1).

## 4. Coherent Electron Transport in DNA

The information that is contained in the base pair sequence of the DNA molecule provides the information by which a nucleic acid Szilard engine (NASE) quantum gate functions because that information is essentially shared between the coherently linked nucleotides and their respective NASE quantum gates. The DNA molecule has been shown to be both a coherent conductor of electrons and a spin filter of electrons, and these two qualities combine to enable concatenation and coherent function of NASE quantum gates. Demonstrated coherent electron conduction occurs longitudinally along the DNA molecule, theoretically because of coherent interactions between the electron pi orbitals of the stacked aromatic nucleotide bases [2,6]. This “pi stacking” provides the coherence necessary to enable electron conduction and/or the potential for the quantum teleportation of an electron’s quantum state. As this coherent conduction or teleportation of electrons takes place it is subject to the spin filtration effects of the DNA molecule. Because of the interaction of electron spin with the helicity of the DNA molecule, an electron that is conducted longitudinally along the helix can be “filtered out” of the helix by the strength of an electromotive force generated by an interaction of the spin of the electron and an inductance-like effect of the DNA helix [7,8]. The strength of the electromotive force generated in such an interaction is dependent in part upon the distance that the electron would be conducted along the DNA helix, and so an electron that is spinning in the appropriate direction would be “filtered out” of the DNA helix at whatever distance would be necessary to develop an electromotive force sufficient for the filtration to occur [3]. An electron that is not spinning in the appropriate direction would not be “filtered out” and would continue along the coherent pathway of the DNA helix until that coherence was broken. The coherence that is provided by the pi stacking of the DNA nucleotide bases is dependent upon the geometric orientation of those bases, and that orientation can be affected by a change in the N-glucosidic bond angle caused by enantiomeric shifting between the C2-endo and C3-endo conformations of the deoxyribose moiety of the nucleotide. Thus, coherence distance and associated electron spin filtration can determine, or be determined by, the conformational shifts in the deoxyribose moiety that are controlled through the NASE quantum gate. Also, the coherence distance and associated electron spin filtration can determine which nucleotide’s NASE quantum gate an electron (or its state) will be deposited (or read) into.

## 5. Measurement and Coherence of Entangled Electrons

The action of an electron passing through the DNA helical spin filter essentially constitutes a “measurement” of the spin of that electron, however, if the distance that that electron travels is not sufficient enough to generate an electromotive force sufficient enough to “filter out” that electron, then no “measurement” is made of that electron’s spin. If that “unmeasured” electron is one member of an entangled pair, and if the second electron member of that entangled electron pair likewise remains “unmeasured”, then the spin states of both electrons in the entangled pair would remain undeclared and the entangled electron pair would maintain its non-local coherence. If the (state of the) first electron of the entangled pair, with its “unmeasured” spin, were to be conducted to and deposited into (or read into) the chirality-determining covalent bond of a nucleotide’s NASE quantum gate, then no determination of chirality would or could be made in that bond because no spin had yet been determined for that electron, and there would be a coherent flexibility in the topology of the DNA molecule at the position of that nucleotide. That flexibility would remain until the second electron of the entangled pair was somehow “measured” and its spin thus “declared”, which would immediately lead to the “declaration” of the spin of the first electron that had been deposited into (or read into) the deoxyribose enantiomeric chirality-determining covalent bond of the nucleotide’s NASE quantum gate, which would bring about a chiral “declaration” in the bond and consequent enantiomeric selection between the C2-endo and C3-endo conformations. The symmetry between the C2-endo and C3-endo enantiomers exists within the function of a logically and thermodynamically reversible Szilard engine, which serves to maintain the coherence of the “undeclared” electron in the chirality-determining covalent bond of the nucleotide’s NASE quantum gate, until that coherence is broken by a measurement of the spin of the other electron of the entangled pair, which would constitute a quantum decision that would bring about a break in the enantiomeric symmetry and “lock” the deoxyribose moiety into either the C2-endo or C3-endo enantiomer.

## 6. Simultaneity of Quantum Logic

The nature of quantum logic is such that the quantum logical processes of DNA occur in simultaneity with each other within and across the coherence of the system until that coherence is broken by a measurement. Thus it is that the double well potential that exists between the C2-endo and C3-endo enantiomeric energy states in each nucleotide provides for the quantum equilibrium that enables multiple NASE quantum gates to simultaneously and coherently mediate multiple potential electron transfers along the spin filtering helix of the DNA molecule. This is a reversible symmetry until a particular enantiomeric selection is determined in a particular NASE quantum gate in a particular nucleotide. The spin filtering that occurs during coherent conduction longitudinally along the DNA molecule provides the measurement that is the quantum decision of enantiomeric selection. Such enantiomeric selection within a system of coherently linked NASE quantum gates constitutes the quantum decision that would bring about the decoherence of that (part of the) system.

## 7. Coherence Stability

The coherence duration time that is appropriate to quantum logic function in DNA is provided by qualities of the DNA itself, and the crystalline nature of DNA provides a coherent stability that was intuited by Schrödinger. As occurs in DNA, the regular and precise design of the nanospace of a crystal limits the degrees of freedom upon which entropic factors such as temperature or solvation can have any effect [13], because if there is only one degree of freedom in a situation then there is zero entropy that can be induced in that situation. Thus the quantum logic in DNA by its nature involves a process of topological change in the DNA molecule, and topological quantum logic by its nature is a fault-tolerant process. These characteristics of the DNA molecule enable extended temporal coherence longitudinally along the DNA molecule and the long-term stabilization of electron spin coherence that occurs through an enantiomeric symmetry at the NASE quantum gate.

The stabilization of the coherence of entangled but “undeclared” electron spin states in the coherent flexibility of the double well potential separating the enantiomeric symmetry between the C2-endo and C3-endo conformations provides for a quantum gate function that, in conjunction with the qualities of DNA that coherently and selectively conduct and spin filter electrons, forms the basis of quantum logic operations in the DNA molecule. The precise design of the nanospace in which this occurs negates entropic effects of temperature and solvation, and thus enables the long-term coherence necessary in biological systems. 

## 8. Conclusions

This paper has presented a heretofore unrecognized model of quantum logic taking place in the DNA molecule that is scalable to large numbers of qubits and occurs at room temperature. Key points of the model are that the DNA properties of the coherent conduction of electrons and the spin filtering of electrons function together simultaneously to selectively read qubits of electron spin into the quantum gate function of an enantiomeric symmetry in the deoxyribose moiety of individual nucleotides that are specifically selected by interaction of helical spin filtration and longitudinal coherence distance along the DNA molecule. Perturbations of the quantum logic system of the DNA molecule can arise from molecules encountered by the system, such as proteins that bind to the DNA molecule, and these perturbations can bring about topological changes in the DNA molecule that are mediated/coordinated through the quantum logic processing capabilities of the DNA molecule. This understanding of biological quantum logic taking place in the DNA molecule can open new vistas of biological understanding, and can provide a conceptual template for architecture of man-made quantum computing.
